# Superbugs-related prolonged admissions in three tertiary hospitals, Kano State, Nigeria

**DOI:** 10.11604/pamj.2019.32.166.18481

**Published:** 2019-04-09

**Authors:** Alkali Bashir, Iliyasu Garba, Adamu Almustapha Aliero, Abdurrazak Kibiya, Muhammad Hassan Abubakar, Ibrahim Ntulume, Faruk Sarkinfada, Agwu Ezera

**Affiliations:** 1 Department of Science Laboratory Technology, School of Technology Kano State Polytechnic, Kano, Nigeria; 2 Department of Microbiology and Immunology, Faculty of Biomedical Sciences Kampala International University Western Campus, Ishaka Bushenyi, Uganda; 3 Department of Medicine, Faculty of Clinical Sciences, Bayero University, Kano State, Nigeria; 4 Department of Microbiology, Faculty of Life Sciences, Kebbi State University of Science and Technology Aliero, Kebbi State, Nigeria; 5 Department of Infection Control, Aminu Kano Teaching Hospital, Kano State Nigeria; 6 Microbiology Unit, Murtala Muhammed Specialist Hospital, Hospital Management Board, Kano State, Nigeria; 7 Department of Medical Microbiology, College of Health Sciences, Bayero University, Kano State, Nigeria; 8 Department of Health and Medical Sciences Khawarizimi International College, Abu Dhabi, United Arab Emirates; 9 Medical Microbiology and Immunology Unit, Department of Biomedical Sciences, Kabale University School of Medicine, Kabale, Uganda

**Keywords:** Superbugs, prolonged admission, tertiary hospitals, Kano

## Abstract

**Introduction:**

Superbugs are pathogenic micro-organism and especially a bacterium that has developed resistance to the medications normally used against it. As the superbug family increases, the need for appropriate diagnostic, treatment, prevention and control strategies cannot be over emphasized. Therefore, this work determined the distribution of superbug bacteria among patients on prolonged hospital admissions in three tertiary hospitals of Kano state, Nigeria.

**Methods:**

A descriptive cross sectional study was undertaken among 401 patients from medical, surgery, orthopedic and burn centre wards in a three tertiary hospitals in Kano state. A sample collected comprises wound/pus, urine, urine catheter and nasal intubation and were analysed using standard microbiological methods for *Acinetobacter* spp and other related nosocomial bacterial pathogens. Antibiotic susceptibility testing was done using Kirby-Bauer disc diffusion method.

**Results:**

One hundred and thirty eight (138) isolates were recovered, from the studied participants. More than 80% of the nosocomial infections (NIs) were caused by Gram-negative bacteria, predominantly Escherichia coli, *Klebseilla* spp, *Proteus* spp, *Pseudomona* spp and *Acinetobacter* spp. In-vitro antibiotic susceptibility test revealed that *acinetobacter* were 100% resistant to amoxicillin, co-trimoxazole, perfloxacin and imipenem.

**Conclusion:**

Superbugs (Acinetobacter species) significantly contributed to delayed hospital admissions through observed 100% resistance to used antibiotics. The healthcare managers of these hospitals and the ministry of health need to take measures against this resistant bacteria (*Acinetobacter* spp) especially on prescribing antibiotics that showed 100% resistant from these studied hospitals.

## Introduction

Antimicrobial resistant poses one of the most pressing public health threats worldwide [1]. The emergence of resistant strains of hospital pathogens has presented a challenge in the provision of good quality of in-patient care. Inappropriate use of antibiotics in the hospital is largely responsible for this problem [2]. Bacterial infections caused by multidrug-resistant (MDR) bacteria are a growing threat worldwide [3]. They are a major cause of morbidity and mortality in developing countries including Nigeria. Antimicrobial resistance (AMR) is a major problem in both hospital and community acquired infections [4, 5]. Several intrinsic factors such as point mutation, gene amplification and extrinsic factors like horizontal transfer of resistant gene between bacteria within and across species by transposes, integrins or plasmids have been postulated for the development of resistance, which cannot be reduced once developed even by restricting the antibiotic usage. Social factors such as demographic changes, poor hygienic practices and overcrowding have been enumerated for the emergence of AMR [6]. Infections caused by resistant bacteria adversely affect treatment outcomes, costs, disease spread and duration of illnesses, posing a serious challenge to the future chemotherapies [5, 7]. In addition to this, the battle between bacteria and their susceptibility to drugs is yet problematic among public, researchers, clinicians and drug companies who are looking for effective drugs [7]. Global burden of antimicrobial resistant (AMR) is still unknown due the lack of comprehensive data from some part of the world especially African countries including Nigeria [1]. But literature has shown that, the burden is at alarming red. For example, among Gram negative bacteria *E. coli* and other unnamed gram negative were reported to have 100% resistant to amoxicillin/clavulanate and chloramphenicol [1]. Nwadike *et al* [8] reported 100% antibiotics resistant of *Acinetobacter baumannii* isolated from the intensive care unit (ICU) of the Nigerian tertiary hospitals to ceftriaxone, amoxicillin-clavulanate, ampicillin-sulbactam, gentamicin, ciprofloxacin and ofloxacin. Resistant of some Gram positive bacteria such as *Staphylococcus aureus* to ampicillin ranged between 0 to 95.6% and coagulate negative showed 100% resistance to ampicillin in some part of the country. Most of the previous research on nosocomial infection and antimicrobial susceptibility from major tertiary hospital in Kano did not focus much on superbugs-related prolonged admissions despite World Health Organisation’s recommendations on frequent research on these organisms [9]. Therefore, this study aimed at determining the superbugs-related prolonged admissions in three tertiary hospitals, Kano state, Nigeria.

## Methods

**Study area:** the study was conducted at Aminu Kano Teaching Hospital (AKTH), Murtala Muhammad Specialist Hospital (MMSH) and Muhammad Abdullahi Wase Specialist Hospital (MAWSH) microbiology laboratory of each hospital, Kano state, Nigeria. The duration of the study was 6 months (July to December 2016).

**Study design:** this was a cross sectional descriptive hospital based which involved isolation of bacteria from urine, wound/pus, urine catheter and nasal feed tube from patients who were eighteen years and above of ages and both sexes admitted in all the above Aminu Kano Teaching Hospital (AKTH), Murtala Muhammad Specialist Hospital (MMSH) and Muhammad Abdullahi Wase Specialist Hospital (MAWSH). The isolated bacteria were subjected to antibiotic sensitivity testing using different antibiotics discs.

**Sample collection:** samples were collected according to the method described by [10]. A sterile open urine container (20ml) calibrated screw-capped was used to collect the urine; a sterile swab stick was used to collect samples from wound/pus, urine catheters, nasal intubation. All samples were labelled accordingly and then transported to the respective laboratory for further analysis. Samples were collected from patients after obtaining ethical approval from the three hospital’s management and verbal informed consent prior to specimen collection from all studied participants. Verbal informed consent was sought from guardians in case of critically ill patient.

**Isolation and identification of bacterial pathogens:** isolation of bacterial agents of urine, wound/pus, urine catheters and nasal tube was done according to the method described by [11, 12]. Four different media were used: 10% blood sheep agar, MacConkey agar and Cysteine Lactose Electron Deficient agar (CLED) and Leeds *Acinetobacter* media. All the media were prepared according to manufacturer's instructions. The media was sterilized at 121°C for 15 minutes. Ten percent (10%) blood agar was prepared by mixing 10ml fresh sheep blood with 90ml of blood agar base at 45°C. Twenty (20ml) of each medium was dispensed into sterile disposable plastic petridish and allowed to set. Each sample was aseptically inoculated (in triplicate) into 10% blood sheep agar, MacConkey agar and Cysteine Lactose Electron Deficient Agar (CLED) and Leeds *Acinetobacter* media. The plates were incubated aerobically at 37°C for 18-24h. The characteristic bacterial isolates observed on the selective media plates were aseptically sub-cultured onto freshly prepared culture media plates. Identification of bacterial isolates was done on the basis of their cultural and biochemical characteristics as described by Cheesbrough [13]. The identified bacterial isolates were maintained on nutrient agar slants stored at 4°C in a refrigerator and subculture periodically.

**Antibiotic susceptibility study:** antibiotic susceptibility was determined using Mueller Hinton (mast group limited). A small inoculum of each pure bacterial isolates was emulsified in 3ml sterile normal saline in a cleaned sterile test tube until it matched with 0.5 McFarland standards. Mueller Hilton agar plates were inoculated using sterile cotton swab in the confluent pattern as in the Kirby-Bauer procedure [14]. Antibiotic discs were placed aseptically on the inoculated plate using sterile forceps. The plates were then incubated for 24h at 37°C. Isolates were considered as sensitive or resistant to an antibiotic according to the diameter of inhibition zone interpretative chart [15]. Antibiotic discs used were: Amoxicillin (AM, 10 μg), Perfloxacin (PEF, 20μg), Ceftriaxone (CTR, 30μg), Ciprofloxacin (CIP, 30μg), Ceftazidime (CAZ, 30 μg), Imipenem (IMP, 10μg), Co-trimoxazole (COT, 30μg), Rocephin (CRO, 10 μg), Augmentin (AMC, 30μg), Tetracycline (T, 30μg). The following standard bacterial isolates: *Escherichia coli* ATCC25922, *Pseudomonas aeruginosa* ATCC27853, *Staphylococcus aureus* ATCC29213 were used as a reference strains for susceptibility test.

**Ethical consideration:** ethical approvals to carry out the study were obtained from Institutional Review Boards of Kampala International University Western Campus, Health Service Management Board Kano (HMB/GEN/488/VOL.1), Ministry of Health Kano State and AKTH, Nigeria. Written informed consent prepared in local languages (Hausa) were obtained from all participants or guardians in case patients were critically ill.

## Results

A total of four hundred and one patients participated in the study. The age of the patients ranged from 18 years to 78 years. Majority of the patients (27.8%) were in the age range of 49-58 years while the 18-28 years age group constituted the least age group (9.2%). There were 200 males and 201 females. Most of the participants were retired (54.9%) while the least were unemployed (3.2%). Most of the participants were married (45.6%) while 1.5% were widows. Out of the 401 studied participants enrolled in this study, 138 (34.42%) bacterial nosocomial pathogens were isolated. Fifty eight (58) bacterial isolates were isolated from AKTH and MMSH each. Twenty two (22) bacterial isolates were obtained from MAWSH. Among the bacterial pathogens isolated, *E. coli* was the most frequently isolated bacterial nosocomial pathogens from all the studied hospitals (Table 1). The results of antimicrobial resistant patterns of nosocomial bacterial pathogens isolated from Aminu Kano Teaching Hospital (AKTH) showed that, *E. coli* showed higher resistance to co-trimoxazole (94%) and less resistance to Augmentin (11%) among all antibiotics tested. *Proteus* spp showed higher resistance to amoxicillin (42%) with less resistance to Imipenem (8%) among all antibiotics tested. *Streptococcus* spp showed 40% resistance to co-trimoxazole, ceftriaxone, rocephin, augmentin, ceftazidime and ciprofloxacin with 0% resistance to tetracycline. *Pseudomnas* spp showed 100% resistance to amoxicillin with 0% resistance to augmentin and imipenem. *Acinetobacter* spp were 100% resistant to amoxicillin, co-trimoxazole, perfloxacin and imipenem with 20% resistant to tetracycline, ciprofloxacin and ceftazidime. *Staphylococcus* spp were 75% resistant to co-trimoxazole and perfloxacin with 25% resistance to tetracycline, ciprofloxacin and ceftazidime. *Klebsiella pneumonie* were resistant 100% resistant to tetracycline with 0% resistance to ciprofloxacin (Table 2).

**Table 1:** Number and percentage of bacterial nosocomial pathogens isolated from the studied participant at three tertiary hospitals in Kano state

Study area/ type of sample	No. examined	No. of positive	Bacterial isolates						
			*Acinetobacter* spp	*Klebsiella* spp	*E. coli*	*Proteus* spp	*Staphylococcus* spp	*Streptococcus* spp	*Pseudomonas* spp
**AKTH**									
Wound/pus	76	17 (29.3)	1(1.3)	0(0)	4(5.3)	2(2.6)	4 (5.1)	3(3.9)	3(3.9)
Urine	51	17 (29.3)	2(3.9)	2(3.9)	9(17.6)	2(3.9)	2(3.9)	0(0)	0(0)
Urine catheter	42	14 (24.1)	2(4.8)	2(4.8)	5(35.7)	4(9.5)	1(2.4)	0(0)	0(0)
Nasal feed tube	31	10 (17.2)	0(0)	3(5)	0(0)	4(6.8)	1(1.7)	2(3)	0(0)
**Total**	**200**	**58 (100)**	**5 (8.6)**	**7(12.1)**	**18(31.0)**	**12(20.7)**	**8(13.8)**	**5(8.6)**	**3(5.2)**
**MMSH**									
Wound/pus	53	21 (36.2)	2(3.7)	1(1.9)	5(9.4)	2(3.7)	4(7.5)	2(3.7)	5(9.4)
Urine	43	20 (34.5)	2(4.6)	6(14.0)	6(14.0)	2(4.6)	2(4.6)	0(0)	2(4.6)
Urine catheter	30	13 (22.4)	1(3.3)	3(10)	2(6.7)	2(6.7)	2(6.7)	2(6.7)	1(3.3)
Nasal feed tube	14	4 (6.9)	1(7.1)	0(0)	0(0)	2(14.2)	1(7.1)	0(0)	0(0)
**Total**	**140**	**58 (100)**	**6 (10.3)**	**10(17.2)**	**13(22.4)**	**8(13.9)**	**9(15.5)**	**4(6.9)**	**8(13.8)**
**MAWSH**									
Wound/pus	27	10 (45.5)	1(4.5)	1(4.5)	2(9)	1(4.5)	2(9)	1(4.5)	2(9)
Urine	16	6 (27.3)	1(4.5)	2(9)	2(9)	0(0)	1(45)	0(0)	0(0)
Urine catheter	10	3 (13.6)	1(4.5)	1(4.5)	1(4.5)	0(0)	0(0)	0(0)	0(0)
Nasal feed tube	8	3 (13.6)	0(0)	0(0)	0(0)	2(9)	0(0)	1(4.5)	0(0)
**Total**	**61**	**22 (100)**	**3(13.6)**	**4(18.2)**	**5(22.7)**	**3(13.6)**	**3(13.6)**	**2(9.1)**	**2(9.1)**

**Key: AKTH:** Aminu Kano Teaching Hospital, **MMSH:** Murtala Muhammad Specialist Hospital, **MAWSH:** Muhammad Abdullahi Wuse Specialist Hospital, **spp:** species.

**Table 2:** Antimicrobial susceptibility of *Acinetobacter* spp and other related bacterial nosocomial pathogens isolated from AKTH

Bacterial isolates	Antibiotics (μg)									
	Amoxicillin10	Co-trimoxazole30	Perfloxacin20	Tetracycline30	Ciprofloxacin30	Ceftazidime30	Augmentin30	Rocephin10	Ceftriaxone30	Imipenem10
	R, n (%)	R, n (%)	R, n (%)	R, n (%)	R, n (%)	R, n (%)	R, n (%)	R, n (%)	R, n (%)	R, n (%)
*E. coli* N=18	16(89)	17(94)	3(17)	16(89)	3(17)	4(23)	2(11)	3(17)	3(17)	5(27)
*Proteus* spp *N*=12	5(42)	3(25)	1(9)	3(25)	2(17)	4(33)	4(33)	4(33)	4(33)	1(8)
*Streptococcus* spp N= 5	1(20)	2(40)	1(20)	0(0)	2(40)	2(40)	2(40)	2(40)	2(40)	1(20)
*Pseudomonas* spp N= 3	3(100)	2(67)	2(67)	2(67)	1(33)	1(33)	0(0)	1(33)	2(67)	0(0)
*Acinetobacter* spp N=5	5(100)	5(100)	5(100)	1(20)	1(20)	1(20)	1(22)	2(40)	2(40)	5(100)
*Staphylococcus* spp N=8	5(63)	6(75)	6(75)	2(25)	2(25)	2(25)	3(37)	2(25)	3(37)	4(50)
*Klebsiella pneumonie* N=7	1(15)	3(43)	1(15)	7(100)	0(0)	4(57)	4(57)	2(29)	2(29)	3(43)

Key: N=total number of organism isolated, n= number of isolates resistant, %= percentage, R=Resistant; AKTH=Aminu Kano Teaching Hospital

The results of antimicrobial sensitivity testing from Muhammad Abdullahi Wuse specialist hospital showed that *E. coli* showed 80% resistance to co-trimoxazole with 0% resistant to imipenem. *Proteus* spp showed 100% resistance to tetracycline with 0% resistant to augmentin and imipenem. *Streptococcus* spp showed 50% resistance to co-trimoxazole, tetracycline, ciprofloxacin, ceftazidime, rocephin and ceftriaxone with 0% resistant to amoxicillin, perfloxacin, augmentin and imipenem. *Pseudomonas* spp were 100% resistant to amoxicillin, tetracycline and ceftazidime with 0% resistant to rocephin. *Acinetobacter* spp were 100% resistant to amoxicillin, co-trimoxazole and perfloxacin with 33% resistant to tetracycline, ciprofloxacin, ceftazidime and cugmentin. *Staphylococcus* spp were 100% resistant to co-trimoxazole, perfloxacin and augmentin with 33% resistant to ceftazidime and ceftriaxone. *Klebsiella pneumonie* showed 100% resistance to tetracycline with 0% resistant to Imipenem (Table 3). The results of antimicrobial sensitivity testing of nosocomial bacterial pathogens isolated from Murtala Muhammad Specialist Hospital, Kano showed that, *E. coli* was 92% resistant to co-trimoxazole and tetracycline with 8% resistance to ciprofloxacin and imipenem. *Proteus* spp were 50% resistant to amoxicillin, co-trimoxazole and ceftriaxone with 12% resistant to tetracycline and imipenem. *Streptococcus* spp were 75% resistant to perfloxacin with 25% resistant to amoxicillin, co-trimoxazole, ceftazidime, augmentin, ceftriaxone and imipenem. *Pseudomonas* spp were 75% resistant to amoxicillin with 0% resistant to imipenem. *Acinetobacter* spp were 100% resistant to co-trimoxazole and perfloxacin with 17% resistant to ciprofloxacin and augmentin. *Staphylococcus* spp had 78% resistant to co-trimoxazole and 22% resistant to perfloxacin, tetracycline and ceftazidime. *Klebsiella pneumonie* was 80% resistant to tetracycline with 10% resistant to amoxicillin (Table 4).

**Table 3:** Antimicrobial susceptibility of *Acinetobacter* spp and other related bacterial nosocomial pathogens isolated from MAWSH

Bacterial isolates	Antibiotics ( μg)									
	Amoxicillin10	Co-trimoxazole30	Perfloxacin20	Tetracycline30	Ciprofloxacin30	Ceftazidime30	Augmentin30	Rocephin10	Ceftriaxone30	Imipenem10
	R, n (%)	R, n (%)	R, n (%)	R, n (%)	R, n (%)	R, n (%)	R, n (%)	R, n (%)	R, n (%)	R, n (%)
*E. coli* N=5	3(60)	4(80)	3(60)	4(80)	1(20)	3(60)	3(60)	2(40)	3(60)	0(0)
*Proteus* spp *N*=3	1(33)	2(67)	2(67)	3(100)	2(67)	1(33)	0(0)	1(33)	2(67)	0(0)
*Streptococcus* spp N= 2	0(0)	1(50)	0(0)	1(50)	1(50)	1(50)	0(0)	1(50)	1(50)	0(0)
*Pseudomonas* spp N= 2	2(100)	1(50)	1(50)	2(100)	1(50)	2(100)	1(50)	0(0)	1(50)	1(33)
*Acinetobacter* sppn=3	3(100)	3(100)	3(100)	1(33)	1(33)	1(33)	1(33)	2(67)	2(67)	2(67)
*Staphylococcus* spp N=3	2(67)	3(100)	3(100)	2(67)	2(67)	1(33)	3(100)	2(67)	1(33)	2(67)
*Klebsiella pneumonie* N=4	1(25)	2(250)	1(25)	4(100)	1(25)	2(50)	1(25)	1(25)	3(75)	0(0)

MAWSH=Muhammad Abdullahi Wase Specialist Hospital

**Table 4:** Antimicrobial susceptibility of *Acinetobacter* spp and other related bacterial nosocomial pathogens isolated from MMSH

Bacterial isolates	Antibiotics (μg)									
	Amoxicillin10	Co-trimoxazole30	Perfloxacin20	Tetracycline30	Ciprofloxacin30	Ceftazidime30	Augmentin30	Rocephin10	Ceftriaxone30	Imipenem10
	R, n (%)	R, n (%)	R, n (%)	R, n (%)	R, n (%)	R, n (%)	R, n (%)	R, n (%)	R, n (%)	R, n (%)
*E. coli* N=13	11(85)	12(92)	3(22)	12(92)	1(8)	3(22)	3(22)	2(15)	3(22)	1(8)
*Proteus* spp *N*=8	4(50)	4(50)	3(37)	1(12)	3(37)	3(37)	2(25)	2(25)	4(50)	1(12)
*Streptococcus* spp N= 4	1(25)	1(25)	3(75)	2(50)	2(50)	1(25)	1(25)	2(50)	1(25)	1(25)
*Pseudomonas* spp N= 8	6(75)	6(75)	5(63)	2(25)	1(12)	2(25)	2(25)	2(25)	2(25)	0(0)
*Acinetobacter* sppn=6	4(66)	6(100)	6(100)	2(34)	1(17)	3(50)	1(17	2(34)	2(34)	2(34)
*Staphylococcus* spp N=9	6(67)	7(78)	7(78)	2(22)	3(33)	2(22)	3(33)	3(33)	3(33)	4(45)
*Klebsiella pneumonie* N=10	1(10)	4(40)	2(20)	8(80)	2(20)	7(70)	3(30)	5(50)	3(30)	4(50)

Key: N=total number of organism isolated, n= number of isolates resistant, %= percentage, R=Resistant, MMSH= Murtala Muhammad Specialist Hospital

## Discussion

More than 80% of the nosocomial infections (NIs) were caused by the Gram-negative bacteria (GNB). *Escherichia coli, Pseudomonas* spp, *Klebsiella pneumonie* and *Proteus* spp and *Acinetobacte* spp constituted more than 80% of the isolates obtained from this study. Increasing importance of GNB in NIs has been reported by previous investigators [8, 16, 17]. This observation was consistent with the findings of this study. Increase in drug resistant bacteria in African healthcare centres makes it difficult for healthcare providers to give effective treatment to the hospitalized patients especially immunocompromised patients. There was lack of comprehensive data in most African healthcare centres to ascertain the magnitude of this problem [1]. This study also determined the antibacterial resistant profile of nosocomial bacteria isolated from HCAIs from three hospitals of Kano state, Nigeria. The results of antimicrobial susceptibility testing from all the three studied hospitals showed that *Acinetobacter* spp were 100% resistant to amoxicillin, co-trimoxazole, perfloxacin antibiotics from AKTHS and MAWSH. Similar result was also obtained from MMSH with exception of amoxicillin which was 66% resistant to *Acinetobacter* spp. This was in line with finding of Nwadike *et al* [8] who reported 100% resistance of *Acinetobacter* spp to amoxicillin-clavulanate and other commonly used antibiotics in a Nigerian tertiary hospital intensive care unit (ICU). Nwadike *et al* [8] added that, there was an increasing report of *Acinetobacter* spp resistant to β-lactams, aminoglycoside antibiotics in many healthcare centres within the country which make some of the healthcare centres a reservoir of *Acinetobacter* spp resistant genes. Odewale *et al* [18] also reported 100% resistance of ciprofloxacin and amikacin from *Acinetobacter* spp isolated in Ladoke Akintola University Teaching Hospital, Osogbo, Nigeria.

The results of this study also found 100% resistance of *Acinetobacter* spp to Imipenem from AKTH. This was in line with findings of Shahcheraghi *et al* [19] who reported 100% of Imipenem from *Acinetobacter* spp from patients at Tehran hospitals, Iran. Other Gram negative bacteria found to be 100% to some of the antibiotics tested from AKTHS and MAWSH were *Proteus* spp, *Pseudomnas* spp and *Klebsiella pneumonie*. The higher resistance of Gram's negative bacteria to antibiotics reported in this could be due to the higher exposure of these bacteria to these antibiotics [8]. Antibiotics susceptibility test results on Gram's positive bacteria isolated from three studied hospital showed that, *Streptococcus* spp showed 0-40%, 0-50% and 0-70% resistant to antibiotics tested from AKTH, MAWSH and MMSH respectively. This was in line with findings of Barma *et al* [20], who reported 7-80% of antibiotics resistant among the coagulase negative staphylococci isolated from Intensive Care Units of the University of Maiduguri Teaching Hospital, Nigeria. *Staphylococcus* spp showed 25-75%, 33-100% and 22-78% resistance to antibiotics tested from AKTH, MAWSH and MMSH respectively. This was in line with finding of Barma *et al* [20] who reported 0-100% antibiotics resistance from *Streptococcus pyogenes* isolated from Intensive Care Units of the University of Maiduguri Teaching Hospital, Nigeria.

## Conclusion

Present study determined the superbugs-related prolonged admissions in three tertiary hospitals, Kano state, Nigeria. The results showed that, *Acinetobacter* spp and other related nosocomial bacterial pathogens were 0-100% resistant to the antibiotics tested. There is need for the management of the three studied hospitals to take measures especially in antibiotics prescription by reviewing guideline to avoid prescribing antibiotics that showed 100% resistance to this bacteria.

### What is known about this topic

- Presence of some nosocomial bacterial pathogens from the three studied hospitals;
- Antibiotic resistant profile of some nosocomial bacterial pathogens from the three studied hospitals.

### What this study adds

- Presence of *Acinetobacter spp* associated with other nosocomial bacterial pathogens from the three studied hospitals;
- Antibiotic resistant profile of *Acinetobacter spp* associated with bacterial nosocomial pathogens from the three studied hospitals.

## Competing interests

The authors declare no competing interests.
